# A Lightweight Dual-Branch Complex-Valued Neural Network for Automatic Modulation Classification of Communication Signals

**DOI:** 10.3390/s25082489

**Published:** 2025-04-15

**Authors:** Zhaojing Xu, Youchen Fan, Shengliang Fang, You Fu, Liu Yi

**Affiliations:** 1 School of Space Information, Space Engineering University, Beijing 101416, China; zhaojing_xu@hgd.edu.cn (Z.X.); love193777@sina.com (Y.F.); 2 Graduate School, Space Engineering University, Beijing 101416, China; you_fu@hgd.edu.cn (Y.F.); yiliu@hgd.edu.cn (L.Y.)

**Keywords:** automatic modulation classification (AMC), complex-valued neural networks (CVNNs), deep learning, Riemannian manifold

## Abstract

Currently, deep learning has become a mainstream approach for automatic modulation classification (AMC) with its powerful feature extraction capability. Complex-valued neural networks (CVNNs) show unique advantages in the field of communication signal processing because of their ability to directly process complex data and obtain signal amplitude and phase information. However, existing models face deployment challenges due to excessive parameters and computational complexity. To address these limitations, a lightweight dual-branch complex-valued neural network (LDCVNN) is proposed. The framework uses dual pathways to separately capture features with phase information and complex-scaling-equivariant representations, adaptively fused via trainable weighted fusion. Spatial and channel reconstruction convolution (SCConv) is extended to complex domain and combined with complex-valued depthwise separable convolution block (CBlock) and complex-valued average pooling to eliminate feature redundancy and extract higher order features. Finally, efficient classification is realized through complex-valued fully connected layers and a complex-valued Softmax. The evaluations demonstrate that LDCVNN achieves the highest average accuracy on RML2016.10a with only 9.0 K parameters and without data augmentation, which reducing the number of parameters by 99.33% compared to CDSN and by 97.25% compared to CSDNN. Additionally, LDCVNN achieves a better balance between efficiency and performance across other datasets.

## 1. Introduction

The rapid advancement of wireless communication technology has exacerbated the scarcity of industrial spectrum resources, driving cognitive radio (CR) technology to become a crucial solution for enhancing spectrum efficiency. As a core functional module of CR systems, automatic modulation classification (AMC) plays a vital role in improving the communication efficiency of Unmanned Aerial Vehicle systems, ensuring reliable interconnection of Internet of Things devices, and enhancing communication anti-interference capabilities [1,2]. Traditional AMC approaches are primarily divided into two categories: statistical inference based on likelihood functions [3,4,5] and machine learning based on handcrafted features [6]. The former is limited by the idealized assumptions of channel models, making it difficult to adapt to complex electromagnetic environments. The latter, although improving practicality through feature engineering such as cyclic spectrum and higher-order cumulants, still relies on expert experience for feature design and environmental adaptability.

In recent years, deep learning-based AMC technology has broken through the limitations of traditional methods, achieving a direct mapping from raw signals to modulation categories through end-to-end feature learning. Mainstream architectures such as convolutional neural network (CNN) [7,8,9], Long Short-Term Memory (LSTM) [10], and their hybrid models [11,12] have demonstrated classification performance surpassing traditional methods on public datasets. However, their underlying real-valued neural networks (RVNNs) have an essential limitation: In-phase and quadrature (IQ) signals have physical coupling in the complex domain, while RVNNs treat them as independent real-number channels, which disrupts the inherent structure of the signal, leading to the loss of phase information and weakened model interpretability.

Meanwhile, complex-valued neural networks (CVNNs) can directly process complex data and achieve collaborative processing of IQ components at the signal representation level. Their advantages are reflected in maintaining the integrity of signal phase-amplitude and possessing rotational equivariance, which can enhance the physical interpretability of modulation features and the robustness of the model to communication interference such as carrier frequency offset.

Complex-valued Neural Networks (CVNNs), initially proposed and theoretically grounded by Clarke and Hirose et al. [13] in 1990, have evolved significantly and have been demonstrated to exhibit potential exceeding that of Real-valued Neural Networks (RVNNs) in handling complex-valued data, particularly in optimization and generalization capabilities [14]. Given the inherent I/Q complex sampling nature of wireless communication signals and the critical role of amplitude and phase information for AMC, CVNNs can directly and effectively process this information, enabling a more complete extraction of waveform features. Consequently, they are considered superior to RVNNs for AMC [15,16,17].

Existing CVNN-based AMC research primarily falls into two main approaches. The first approach is based on the key complex components proposed by Trabelsi et al. [14], which involves extending layers of RVNNs to implement complex-valued operations, effectively achieving a transition from the real domain to the complex domain. For instance, Li et al. [18] proposed a complex-valued DNN, which exhibits lower error rates and fewer parameters compared to real-valued DNNs and complex-valued ResNets. Tu et al. [19] systematically compared the performance of three complex-valued neural networks with their equivalent real-valued counterparts for AMC. The results indicated that complex-valued neural networks generally outperform real-valued neural networks at higher Signal-to-Noise Ratios (SNRs) and can extract useful features from signals earlier at lower SNRs. Although the computational complexity of complex-valued neural networks is typically higher than that of real-valued neural networks, the computational cost can be brought closer to that of real-valued neural networks by adjusting the number of parameters. In 2022, to further improve the performance of CVNN-based AMC models, S. Kim et al. [20] extended max-pooling and Softmax functions to the complex domain and developed complex-valued CNN and complex-valued ResNet accordingly. Comparative experiments with real-valued CNNs and ResNets demonstrated that the proposed complex-valued classifiers significantly improved the performance of signal modulation recognition, particularly enhancing the recognition accuracy of phase-related modulation types. Furthermore, Xu et al. [17] proposed complex-valued VGG and complex-valued ResNet architectures and found that CVNNs offer higher accuracy and fewer model parameters compared to RVNNs with equivalent network architectures. In 2024, Cheng et al. [21] utilized complex-valued convolution layers, dropout layers, and complex average pooling layers to construct multiple local feature learning blocks, aimed at learning the local and hierarchical correlations of IQ signals, and combined CNN and LSTM to achieve higher precision classification. Despite the effectiveness of these methods in leveraging the amplitude and phase information of signal data, they exhibit sensitivity to complex-valued scaling. This implies that the extracted features are prone to significant changes with variations in the amplitude of the input signal, leading to a certain degree of loss in signal content and potentially affecting the robustness of the algorithm in some scenarios. While data augmentation (DA) techniques can be used to train the model to mitigate this issue, such external data processing methods are not only time-consuming but also offer limited effectiveness.

Another approach, proposed by Chakraborty et al. [22,23], is based on Riemannian manifold theory. This approach models the complex domain as a product manifold of scaling and rotation groups and defines convolution and distance transformations on the manifold, enabling the model to effectively ignore variations caused by channel attenuation or clock skew, thereby significantly enhancing the robustness of automatic modulation recognition. This method features a smaller model size, high computational efficiency, and excels in data classification and information extraction. However, it is worth noting that this approach discards a significant amount of phase information, and due to the limitations of the SurReal framework in handling complex algebraic operations, its performance on large datasets may not be ideal [24].

Additionally, considering the limited computational resources often encountered in real-world industrial scenarios, the large model size and high computational complexity can hinder the deployment of the model on hardware. Consequently, model lightweighting has gradually become a focal point of research in this field. Generally, AMC methods based on real-valued neural networks reduce computational costs through model compression (e.g., channel pruning [25,26,27]), architecture search [28,29] (e.g., MobileNet), or knowledge distillation [30]. However, these methods do not consider the characteristics of the complex domain, and directly migrating them to CVNNs may lead to a significant decline in performance. Some researchers have adopted lightweight real-valued neural networks as the main architecture, only replacing local modules with complex components, and combining them with data augmentation techniques to balance accuracy and computational complexity. For example, Wang et al. [31] combined complex-valued separable convolution and residual networks and added a hybrid data augmentation technique of rotation and splicing to compensate for the potential performance loss caused by lightweight design. And Guo et al. [32] proposed a lightweight convolutional neural network by combining data augmentation, complex-valued convolution, depthwise separable convolution(DSC), channel attention mechanisms, and channel shuffling, achieving a lightweight and low-complexity automatic modulation classification with only 9751 model parameters. In addition, Xiao et al. [33] attempted to extend DSC to the complex domain and combine it with residual connection structures to construct a lightweight structure suitable for CVNNs, aiming to facilitate the lightweight deployment of AMC systems.

Therefore, most existing CVNNs for AMC focus on performance improvement while neglecting a critical constraint in practical deployments: industrial-grade equipment’s stringent requirements for model lightweighting and computational efficiency. And current research still lacks a lightweight AMC framework specifically designed for the characteristics of the complex domain. Most current CVNNs fail to meet data processing demands in resource-constrained scenarios due to complex arithmetic units and redundant parameter designs.

To address the aforementioned issues, a lightweight dual-branch complex-valued neural network (LDCVNN) is proposed, which achieves efficient deployment while ensuring the advantages of complex-domain processing. The main innovations include:

- To integrate the advantages of the two main approaches in existing CVNN-based AMC, a dual-branch extraction structure is designed to capture both features containing rich phase information and features with complex-scaling equivariance. These features are then fused using a trainable weighted fusion.
- To reduce redundant complex-valued features of CVNNs, spatial and channel reconstruction convolution (SCConv) is extended to the complex domain;
- To further enhance feature diversity and facilitate efficient feature mining and dimensionality reduction, the fused features are further extracted by complex-valued spatial and channel reconstruction convolution (CSCConv), complex-valued depthwise separable convolution block (CBlock) and Complex-valued average pooling (CAP).

## 2. Methodology

This section can be divided into three parts. The first part focuses on the problem statement and the signal model. The second part elaborates on the design and implementation of the complex-valued modules. And the third part provides a detailed introduction to the model proposed in this paper.

### 2.1. Problem Statement and Signal Model

AMC serves as a key technology in cognitive radio systems, playing an important role in dynamic spectrum access. Its primary task is to analyze the time–frequency characteristics of received signals to accurately identify different modulation types and transmitter types, thereby enabling dynamic spectrum allocation and interference avoidance. This technique establishes a mapping relationship between the feature space of received signals and the modulation category space, providing fundamental support for spectrum sensing and decision-making.

The input signal is a complex-valued baseband time series obtained by sampling the in-phase and quadrature components of the radio frequency signal. Since tasks like modulation classification require real-valued outputs, automatic modulation classification for wireless communication signals can be regarded as a multi-classification problem that maps complex-valued inputs to real-valued outputs: f∈F:S→C. Here, S and C represent the sample space and category space, respectively. Considering practical requirements, deep learning networks approximate the mapping function between S and C by learning the relationships between the data and their corresponding labels in the dataset. The optimization objective can be expressed as follows:

(1) $$\underset{f\in F}{\min}E_{(s,\mathrm{label})∼D}\{L_{ce}[f(s),\mathrm{label}]\}$$

Here, D represents the existing dataset, label denotes the modulation category labels of signal data s, and $L_{ce}$ is the cross-entropy loss function.

Signals in the sample space can be either continuous or composed of discrete modulated bits, containing factors such as variations in frequency, phase, or amplitude. Additionally, the signals may be affected by path loss and superimposed with Gaussian white noise representing thermal noise. Therefore, the equivalent complex baseband signal model with in-phase and quadrature components used in this paper is as follows:

(2) $$s(l)=A(l)e^{j(\omega l+\varphi )}x(l)+n(l),l=1,\ldots ,L$$

In the equation, s(l) represents the received signal stored in the form of discrete in-phase and quadrature components, with a sample length of L, A(l) denotes the wireless channel gain, x(l) represents the transmitted signal, and n(l) represents the complex additive Gaussian white noise. For convenience in processing satellite communication signal data and performing modulation recognition, the received signal s can be expressed as:

(3) $$s=\left[\begin{array}{c} ℜ\{s[1]\},\ldots ,ℜ\{s[L]\}\\ ℑ\{s[1]\},\ldots ,ℑ\{s[L]\} \end{array}\right]=\left[\begin{array}{c} S_{I}\\ S_{Q} \end{array}\right]=S_{I}+jS_{Q},\begin{array}{cc} & S_{I}, \end{array}S_{Q}\in \mathbb{R}$$

Here, the received signal s is represented as a complex vector where each element is split into its real part ℜ{s[l]} and imaginary part ℑ{s[l]}. $S_{I}$ and $S_{Q}$ represent the in-phase component and quadrature component obtained after down-conversion of the signal, and j is the imaginary unit. From a mathematical perspective, the I/Q component correspond to the real and imaginary parts, respectively, and there is a mapping relationship between them during each multiplication operation, which is often overlooked in most deep learning-based AMC models. Additionally, the amplitude $\left|s\right|$ and phase ∠s, which contain the information from the in-phase and quadrature channels, can be expressed as follows:

(4) $$\left|s\right|=\sqrt{S_{I}^{2}+S_{Q}^{2}}$$

(5) $$∠s=\arctan(S_{Q},S_{I})$$

### 2.2. Complex-Valued Building Blocks

#### 2.2.1. Complex-Valued Convolution (CConv)

To directly process complex-valued data and avoid information loss or redundancy, complex-valued convolutions are employed. The complex convolutional kernel could be defined as $k=k_{r}+jk_{i}$, and for complex-valued input $s=S_{I}+jS_{Q}$, real-valued operations are used to simulate complex-valued operations. The complex-valued convolution could be obtained using the formula shown below [14]:

(6) $$\mathrm{CConv}(s)=k*s=(k_{r}*S_{I}-k_{i}*S_{Q})+j(k_{r}*S_{Q}+k_{i}*S_{I})$$

Here, ∗ denotes the point-wise multiplication operation. Although this approach achieves an extension from the real domain to the complex domain, allowing for full utilization of the amplitude and phase information in the IQ signals, the extracted features exhibit sensitivity to complex-valued scaling. In certain scenarios, this sensitivity may affect the robustness of the algorithm.

To address this issue, the weighted Fréchet mean filtering [34] is simultaneously employed as convolution to process the IQ data of the signal [22]. This ensures the preservation of geometric structural features, such as phase relationships and amplitude ratios, enabling the model to ignore changes caused by channel attenuation or clock offsets without requiring additional learning of features at different scales or phases. The objective of weighted Fréchet mean (wFM) convolution is to find a complex number m such that the sum of squares of the weighted distances from all input points to that point is minimized. This definition not only retains the weighted average characteristic of traditional convolution, but also takes into account the special properties of complex-valued data.

From the perspective of manifold geometry, the non-zero complex plane $\tilde{C}$ can be viewed as the product manifold of positive magnitudes and planar rotations. By modeling the complex domain as the product manifold of the scaling group $R^{+}$ and the rotation group SO(2), the distance metric between any two complex-valued data $s_{1},s_{2}\in \tilde{C}$ could be defined as [22]:

(7) $$d\left(s_{1},s_{2}\right)=\sqrt{\log^{2}\frac{\left|s_{2}\right|}{\left|s_{1}\right|}+\log{\left\|R\left(∠s_{2}\right)R{\left(∠s_{1}\right)}^{-1}\right\|}^{2}}$$

(8) $$R(∠s(l))=\left[\begin{array}{cc} \cos(∠s(l)) & -\sin(∠s(l))\\ \sin(∠s(l)) & \cos(∠s(l)) \end{array}\right],l=1,\ldots ,L$$

For a signal sequence s(l) of length L, R(∠s(l)) is a 2 × 2 rotation matrix of $R^{+}\times \mathrm{SO}(2)$ corresponding to the phase ∠s(l) of the signal sequence at time step l. The wFM convolution kernel on this manifold is obtained by optimizing the weighted variance of the manifold distances [22]:

(9) $$\begin{array}{c} \mathrm{wFM}\left(\left\{w_{k}\right\},\left\{s_{k}\right\}\right)=\arg\underset{m\in \tilde{C}}{\min}\sum_{k}w_{k}d^{2}\left(s_{k},m\right) \end{array}$$

The weight $\left\{w_{k}\right\}$ satisfies the convex combination constraint to ensure the closure of the manifold.

#### 2.2.2. Complex-Valued Activations

The rectified linear unit (ReLU) is primarily used to capture the nonlinear features in the data. The complex-valued ReLU is implemented by applying a real-valued activation function separately to the real and imaginary parts of the features, and its expression is given in Equation (10) [14]. The Tangent ReLU [22] (tReLU) is equivalent to implementing the ReLU operation on a Riemannian manifold. It projects the manifold points onto the tangent space via a logarithmic map, applies the ReLU activation in the tangent space, and then maps the result back to the manifold using an exponential map. This operation preserves the manifold structure while achieving feature sparsification and enhancing the nonlinear expressive capability. The formula is presented as Equation (11).

(10) CReLU(s)=ReLU(SI)+jReLU(SQ)

(11) tReLU(s)=exp(ReLU(logs)+jReLU(∠s))

#### 2.2.3. Complex-Valued Max Pooling (CMP)

Complex-Valued Max Pooling (CMP) resolves this by comparing magnitudes through amplitude-phase optimization. The formula is as follows:

(12) $$\left\{\begin{array}{c} I=Index({\mathrm{MP}}_{k,s}\left\{\left|s\right|\right\})\\ {\mathrm{CMP}}_{k,s}(s)=S_{I}(I)+jS_{Q}(I) \end{array}\right.$$

Here, k and s denote the pooling kernel size and stride, respectively. I represents the index matrix of the maximum value after local regional max-pooling, and MP refers to the real-valued max-pooling operation.

#### 2.2.4. Complex-Valued Average Pooling (CAP)

Complex-valued average pooling can be achieved by calculating the average of the real and imaginary parts of all complex numbers within a local region separately. The formula for CAP is as follows:

(13) $${\mathrm{CAP}}_{k,s,p}(s)={\mathrm{AP}}_{k,s,p}(\left|s\right|)$$

Here, k, s and p, respectively denote the pooling size, stride, and padding size, while AP represents the real-valued average pooling operation.

#### 2.2.5. Complex-Valued Batch Normalization (CBN)

The mathematical expression for the complex-valued batch normalization is given in the following formula [14]:

(14) $$\mathrm{CBN}(\tilde{s})=\gamma \tilde{s}+\beta$$

Here, γ and β are the scaling parameter and shifting parameter in the complex domain, respectively, and $\tilde{s}$ is the normalized representation of the complex-valued input s.

(15) $$\gamma =\left[\begin{array}{cc} \gamma_{rr} & \gamma_{ri}\\ \gamma_{ir} & \gamma_{ii} \end{array}\right]$$

(16) $$\tilde{s}={\left(V\right)}^{-\frac{1}{2}}(s-E(s))$$

(17) $$V=\left[\begin{array}{cc} \mathrm{cov}(S_{I},S_{I}) & \mathrm{cov}(S_{I},S_{Q})\\ \mathrm{cov}(S_{Q},S_{I}) & \mathrm{cov}(S_{Q},S_{Q}) \end{array}\right]$$

Here, the scaling parameter γ consists of $\gamma_{rr}$ and $\gamma_{ii}$, both initialized to 1, while $\gamma_{ir}$ and $\gamma_{ri}$ as well as the real and imaginary parts of the complex-valued offset β are all initialized to 0. ${\left(V\right)}^{-\frac{1}{2}}$ is the square root of the inverse of the input covariance matrix V, and $\tilde{s}$ follows a standard complex-valued distribution with a mean of 0, covariance of 1, and pseudo-covariance of 0. cov(⋅,⋅) is the covariance operator.

#### 2.2.6. Complex-Valued Full Connection (CFC)

Unlike simple linear transformations, the complex-valued full connection (CFC) performs a combination of the real and imaginary components of neurons. $w_{r}$ and $w_{i}$ are the real and imaginary parts of the complex-valued fully connected weight, respectively.

(18) $$\mathrm{CFC}(s)=(w_{r}\times S_{I}-w_{i}\times S_{Q})+j(w_{r}\times S_{Q}+w_{i}\times S_{I})$$

#### 2.2.7. Complex-Valued Depthwise Separable Convolution Block (CBlock)

**Figure 1:**
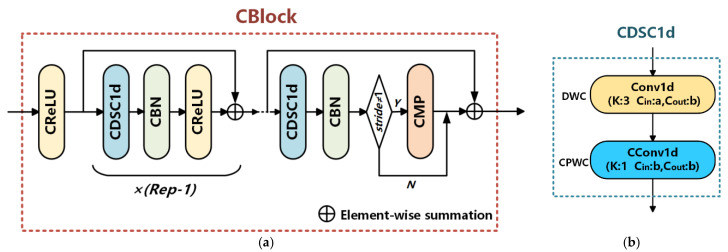
Schematic diagrams of Complex-Valued Depthwise Separable Convolution Block (CBlock). (**a**) The structure of the CBlock; (**b**) The structure of the one-dimensional complex-valued depthwise separable convolution (CDSC1d).

The composition of the complex-valued depthwise separable convolution block (CBlock) [33] is illustrated in Figure 1a. Its structure includes residual connections, where all convolution layers in the residual paths are one-dimensional, complex-valued depthwise separable convolutions (CDSC1d) [33]. With its unique architecture, CBlock can effectively capture the intrinsic relationships in complex-valued data using a lightweight design, thereby reducing computational complexity while enhancing the model’s expressiveness and performance. Rep denotes the number of repetitions, and stride represents the stride value. The CDSC1d leverages the mapping advantages of depthwise separable convolution (DSC) to simplify cross-channel and spatial correlations. It is implemented through complex-valued pointwise convolution (CPWC) with complex-valued operations on the channel dimension and depthwise convolution (DWC) with real-valued operations on the spatial dimension, as shown in Figure 1b.

#### 2.2.8. Complex-Valued Spatial and Channel Reconstruction Convolution (CSCConv)

Spatial and channel reconstruction convolution (SCConv) [35] is an efficient convolution module composed of a spatial reconstruction unit (SRU) and a channel reconstruction unit (CRU), which are designed to suppress spatial redundancy and reduce channel redundancy, respectively. To adapt SCConv for CVNNs in handling complex-valued features, we made innovative improvements. Specifically, the SRU was retained due to its proven effectiveness in evaluating and reconstructing spatial features, as well as its strong performance in suppressing spatial redundancy and enhancing feature representation. However, the CRU, while effective at reducing channel redundancy for real-valued features using the Split-Transform-and-Fuse strategy, exhibits limitations when dealing with complex-valued features. Therefore, we replaced the original CRU with a complex-valued channel reconstruction unit (CCRU), resulting in the proposed complex-valued spatial and channel reconstruction convolution (CSCConv).

**Figure 2:**
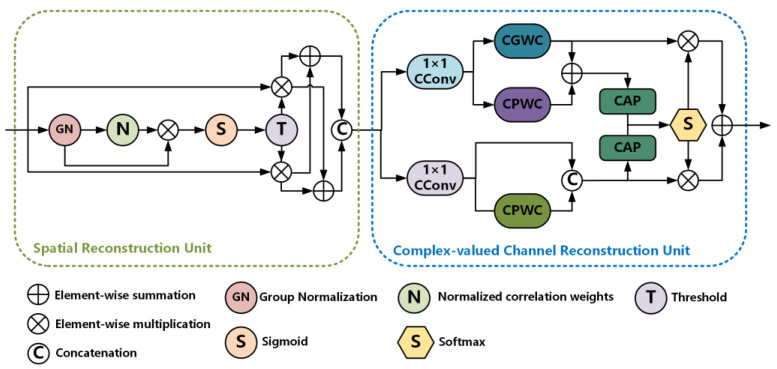
The structure of Complex-Valued Spatial and Channel Reconstruction Convolution (CSCConv).

The structure of the SRU is illustrated in the green dashed area of Figure 2. It uses the scaling parameters of the group normalization (GN) layer to measure spatial information across channels, calculates normalized weights to generate reweighting factors for enhancing or suppressing different parts of the feature map, and performs grouping and recombination operations to reconstruct spatial features. This process simplifies spatial information processing and improves computational efficiency.

The architecture of the CCRU is shown in the blue dashed area of Figure 2. During the data splitting stage, the input is divided into two parts, and channel-wise compression is performed via 1 × 1 CConv to ensure full utilization of complex-valued information in subsequent processing. In the transformation stage, complex-valued groupwise convolution (CGWC) and CPWC are introduced to more effectively extract high-level information from complex-valued features while reducing computational redundancy. Both CGWC and CPWC replace traditional two-dimensional real-valued convolutions with one-dimensional CConv layer. In the fusion stage, complex global average pooling is applied to reduce feature dimensions in the complex domain while preserving statistical information of complex-valued features, facilitating subsequent feature fusion. Other settings remain consistent with those of Reference [35].

#### 2.2.9. Complex-Valued Softmax (CSoftmax)

Typically, Softmax is used as the final step in deep learning-based AMC models to normalize predictions into a probability distribution. In this paper, the real-valued Softmax is extended to the complex domain by utilizing the magnitude of complex data [20].

(19) $$CSoftmax(s)=\frac{\exp(\left|s\right|)}{\sum_{m=1}^{n}\exp(\left|s_{m}\right|)}$$

### 2.3. Proposed Model

**Figure 3:**
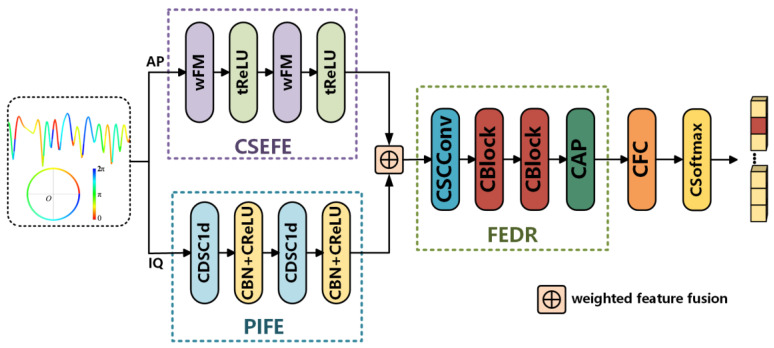
The structure of the proposed LDCVNN.

In order to balance computational complexity and classification accuracy, a lightweight dual-branch complex-valued neural network (LDCVNN) is proposed for automatic modulation classification (AMC) of communication signals. The structural diagram of LDCVNN is shown in Figure 3. First, the original IQ signals are processed by the phase information feature extraction (PIFE) module and the complex-valued scaling equivariant feature extraction (CSEFE) module, respectively, to extract features rich in phase information and features with complex scaled equivariance. Then, the trainable weighted feature fusion is used to adaptively adjust the weights, thereby effectively fusing the features extracted from the two branches. Subsequently, the feature extraction and dimension reduction (FEDR) module is used to achieve more efficient feature mining and dimension reduction. Finally, the extracted features are mapped to the modulation category space through a CFC and a CSoftmax, completing end-to-end automatic modulation classification.

The specific design of each functional module is described below:

PIFE Module: This module is constructed by cascading CDSC1d, CBN, and CReLU. The PIFE module aims to effectively capture the instantaneous phase characteristics of the signal and retain the joint spectral-temporal information.

CSEFE Module: This module is based on Riemannian manifold geometry theory and is implemented using a cascaded structure of wFM layer and tReLU layer. This module first converts original IQ signals into Amplitude-Phase (AP) signals and then extracts features with complex-valued scaling equivariance through orthogonal decomposition in the manifold space.

FEDR Module: This module comprises CSCConv, CBlock and CAP. This module aims to further extract high-level semantic features, enhance feature diversity, reduce redundant computation, and ultimately achieve efficient feature mining and dimension reduction.

## 3. Experiments

### 3.1. Datasets and Experimental Condition

Table 1 shows the datasets used in the experiments. Among these, RML2016.10a and RML2016.10b simulate time-varying stochastic channel effects under adverse propagation conditions, such as additive white gaussian noise and frequency offsets [36,37]. The RML2018.01a is collected indoors using USRP B210 at the 900 MHz ISM frequency band [38]. HisarMod2019.1 includes a wider range of multipath fading types (ideal, static, Rayleigh, Rician, and Nakagami-m channels) with different delays, making it more representative of real-world wireless communication environments [39]. A total of 156,000 samples (300 signals × 26 modulation categories × 20 SNR levels) are selected from HisarMod2019.1 for experimentation. Table 2 presents the detailed layer specifications of the proposed model under different data lengths. SNR refers to the in-band Signal-to-Noise Ratio. The in-band SNR is defined as the ratio of the desired signal power to the noise power within the signal bandwidth.

In the experiments, the cross-entropy loss function is adopted, and the Adam optimizer is used for model training. The initial learning rates are set to 0.01 and 0.001, respectively, with a total of 300 training epochs and a batch size of 400. Other hyperparameters are kept at their default values. Two training strategies are employed during the training process: one involves dynamically adjusting the learning rate, where the learning rate is halved if the validation accuracy does not improve over five consecutive epochs; the other maintains a fixed global learning rate throughout the training. After training, the model parameters with the highest classification accuracy on the validation set are selected as the optimal parameters for testing the model’s classification accuracy on the test set. The experiments are conducted using a GeForce GTX 3090 GPU, an Intel(R) Xeon(R) Gold 6248R CPU, the Windows 10 operating system, and the deep learning framework is PyTorch 1.11.0.

### 3.2. Evaluation Method

According to the true values and predicted values, the entire sample set can be divided into true positive (TP), false positive (FP), true negative (TN), and false negative (FN). Therefore, accuracy is defined as the proportion of all correctly classified samples to the total number of samples. F1-score is a harmonic average of precision and recall that allows for a comprehensive evaluation of model performance. In the experiments, accuracy and F1-score are used to evaluate the performance of the network model on different datasets. Accuracy and F1-score could be calculated as follows:

(20) $$\mathrm{Accuracy}=\frac{TP+TN}{TP+TN+FP+FN}$$

(21) $$\left\{\begin{array}{c} F_{1}\text{-}\mathrm{score}=\frac{2\times \mathrm{Precision}\times \mathrm{Recall}}{\mathrm{Precision}+\mathrm{Recall}}\\ \mathrm{Precision}=\frac{\mathrm{TP}}{\mathrm{TP}+\mathrm{FP}}\\ \mathrm{Recall}=\frac{\mathrm{TP}}{\mathrm{TP}+\mathrm{FN}} \end{array}\right.$$

Here, precision indicates the proportion of samples that are actually positive among all the samples predicted as positive by the model. And recall indicates the proportion of actually positive samples that are correctly predicted as positive by the model. The core goal of model lightweighting is to reduce resource consumption and enhance performance in edge computing. Therefore, it is crucial to comprehensively evaluate the degree of model lightweighting. This paper conducts a quantitative analysis of the model from multiple dimensions, including inference speed, floating-point operations per second (FLOPs), and the number of parameters.

The number of model parameters refers to the total count of all trainable parameters, reflecting the model’s ability to learn and store information. A higher parameter count enhances the model’s expressive power but also increases the risk of overfitting, requiring more data for training and greater computational resources; inference speed is defined as the average time required by the model to process a single sample during the testing phase, which is an important metric for measuring model efficiency; and FLOPs represents the amount of floating-point operations required for a single forward pass of the model. Lower FLOPs indicate higher computational efficiency and a more lightweight model design.

### 3.3. Experiment Settings

**Figure 4:**
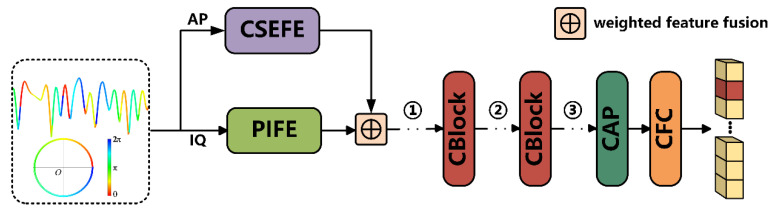
The positions of CSCConv.

As a preliminary step to evaluating model performance, we performed a pre-experiment to determine the most effective position for the CSCConv within the LDCVNN. CSCConv is an efficient convolutional module designed to effectively extract channel features of the target while reducing redundant computations. When placed in an appropriate position, it could help the model learn faster and converge more effectively during training. To investigate the optimal placement of CSCConv in the LDCVNN, experiments were conducted by placing CSCConv at three different positions as shown in Figure 4, marked as ①, ②, and ③. In addition, to assess the influence of the number of repetitions of the first CBlock on the overall model performance, the experiment was conducted with repetition counts ranging from 1 to 8. Furthermore, the model design exhibiting the best results in preliminary experiments was then used as the basis for all subsequent ablation and comparative analyses. The RML2016.10a was used for the experiments, with a training/validation/testing split of 6:2:2.

Ablation studies were performed on the RML2016.10a. Random sampling without replacement was used to create a 6:2:2 split for the training, validation, and testing sets. The LDCVNN was then compared against LDCVNN-A, LDCVNN-B, and LDCVNN-C to validate the effectiveness of each module within LDCVNN for AMC. Here, LDCVNN represents the baseline model without any ablation modifications. LDCVNN-A, LDCVNN-B, LDCVNN-C, and LDCVNN-D represent versions of LDCVNN where the CSEFE, PIFE, CSCConv, or the second CBlock were removed, respectively. LDCVNN + DA represents the proposed LDCVNN model augmented with a data augmentation (DA).

Comparative experiments were conducted on the RML2016.10a, RML2016.10b, RML2018.01a, and HisarMod2019.1. Random sampling without replacement was initially applied to create training, validation, and testing set splits with ratios of 6:2:2, 6:2:2, 3:4:4, and 1:1:1, respectively. To evaluate the generalization ability and lightweight characteristics of the LDCVNN, several key AMC models were selected as benchmarks for comparison, as presented in Table 3. During the training process, the initial learning rate for CDSN was set to 0.0001, which was referenced from the Reference [19]. All other parameter settings remained consistent with those described in Section 3.1.

## 4. Results and Discussions

### 4.1. Pre-Experiment

**Figure 5:**
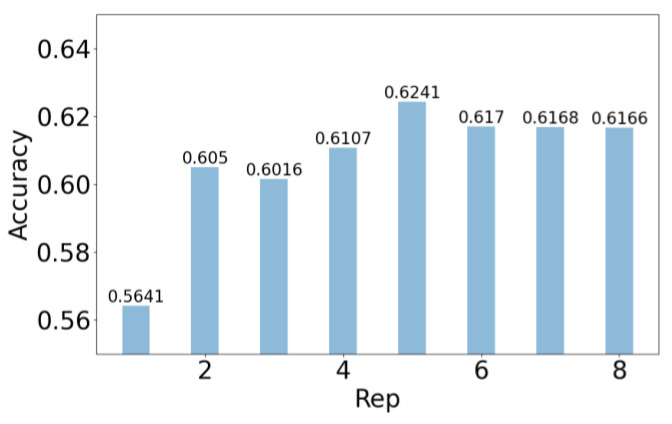
Effect of the repetition counts of the first CBlock.

The experimental results in Table 4 demonstrate that Position ① achieves the optimal accuracy with the fewest model parameters. This suggests that placing the CSCConv after feature-weighted fusion and before the CBlock helps to reduce redundant information in the intermediate layers, thus more effectively facilitating subsequent deep feature learning. Figure 5 demonstrates that the model attains its peak accuracy on the test set when the repetition count is set to 5.

### 4.2. Ablation Experiments

Table 5 presents the average accuracy and model’s parameters of the LDCVNN after ablation studies and with the addition of a DA module. The average accuracy (all SNRs) represents the accuracy averaged over all SNRs and all modulation categories. The results are evident that the model’s accuracy slightly decreases following the ablation operations, while the model capacity also reduces. Among the removed components, the exclusion of PIEF has the most significant impact on model accuracy, resulting in a 6.18% decrease. This indicates that PIEF module plays a critical role in modulated signal classification within the model, corroborating its ability to extract complex features rich in phase information, which is essential for AMC tasks. The removal of CSCConv, the second CBlock, or the CSEFE module leads to accuracy drops of 1.61%, 0.88%, and 0.52%, respectively. These findings suggest that these modules contribute to model accuracy without significantly increasing computational burden, even when the baseline accuracy is already relatively high. Their effectiveness is further demonstrated by their cooperative interactions with other modules, enhancing classification accuracy. This validates the effectiveness of each module in improving the model’s performance for AMC.

Additionally, compared to LDCVNN, the classification accuracy of LDCVNN + DA improves by only 0.05%. This result demonstrates that the proposed LDCVNN model can effectively retain the phase information of the original data while preserving the complex-scaling equivariance of complex features, enabling it to capture important characteristics of IQ signals. Consequently, the model does not rely on data augmentation to enhance performance during training. Furthermore, this suggests that the LDCVNN model exhibits strong robustness in handling data, adapting well to the existing training dataset without requiring additional transformations.

### 4.3. Comparative Experiments

**Figure 6:**
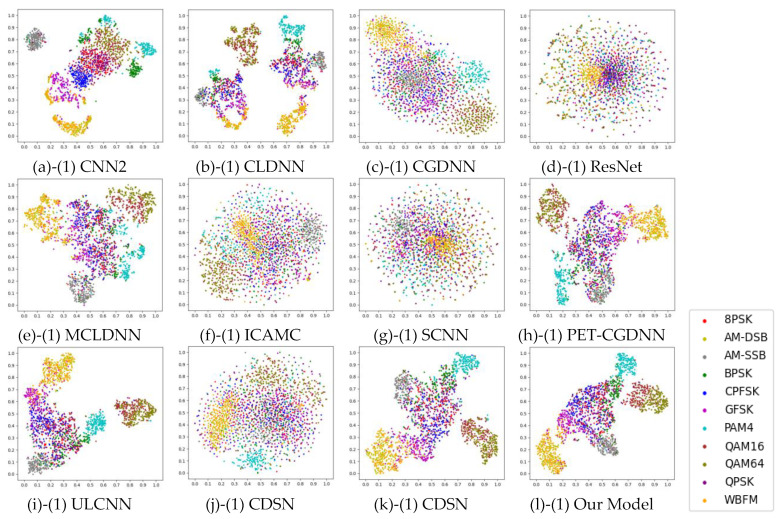
T-SNE Visualization of Model Feature Distributions on RML2016.10a at SNR = −6 dB.

**Figure 7:**
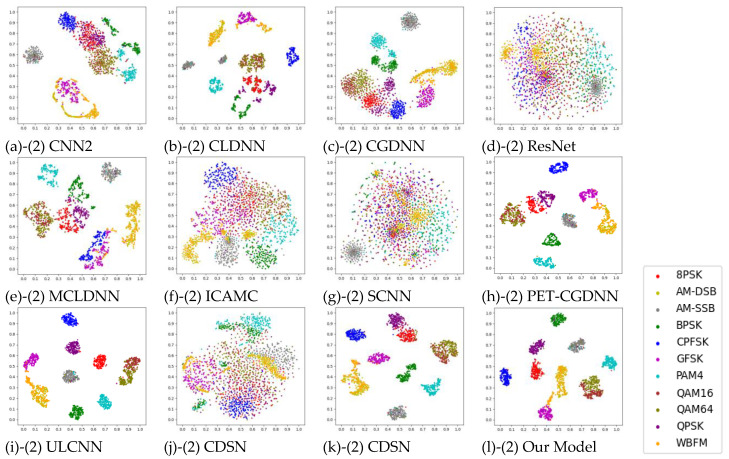
T-SNE Visualization of Model Feature Distributions on RML2016.10a at SNR = 2 dB.

Table 6, Table 7, Table 8 and Table 9 summarizes the performance of our model compared to various benchmark models on four datasets—RML2016.10a, RML2016.10b, RML2018.01a and HisarMod2019.1. Average Accuracy (≥0 dB) represents the average accuracy when the SNR is greater than or equal to 0 dB. Highest accuracy represents the maximum average accuracy at various SNRs. Figure 6 and Figure 7 present t-SNE visualizations of the feature distributions for various models on RML2016.10a at different SNR level. The features, extracted before the classifiers of each model, are projected into a two-dimensional coordinate system after dimensionality reduction using t-SNE. As illustrated in Figure 6 and Figure 7, specific modulation categories are represented by different colors. These visualizations demonstrate the data distribution of each modulation category and the separation degree between different categories.

In terms of model’s parameters, our model exhibits a significant advantage, maintaining a stable parameter size of 9.0–11.3 K on all datasets. This represents a three-order reduction compared to ResNet (21,450 K). As shown in Table 6, our model outperforms other lightweight models such as ULCNN (9.4 K) and CSDNN (327.1 K) on RML2016.10a, demonstrating superior parameter efficiency. This efficiency stems from the collaborative optimization and lightweight design of the modules within the proposed model, which effectively minimizes parameter redundancy.

Regarding computational complexity, our model achieves extremely low FLOPs of 0.00060 G/Sample on RML2016.10a, which is only 73% of the similarly lightweight ULCNN (0.00082 G/Sample) and three orders of magnitude lower than the computationally intensive ResNet (0.993 G/Sample). This low computational cost makes the model highly suitable for resource-constrained environments such as edge devices and real-time communication systems.

In terms of performance, our model achieves average accuracies of 62.41% and 63.97% on RML2016.10a and RML2016.10b, respectively, surpassing traditional models like CNN2 (51.46%/48.74%) and lightweight models like ULCNN (60.58%/63.09%). Additionally, its performance is comparable to the more computationally expensive MCLDNN (60.83%/65.49%), with a gap of less than 1.5%. This indicates that the lightweight design of our model does not compromise its core classification capabilities. The F1-Score trends align with the accuracy results, confirming that the model achieves a balanced trade-off between precision and recall. Meanwhile, our model demonstrates strong performance on RML2018.01a and HisarMod2019.1, which have larger data length, achieving slightly higher average and high accuracies compared to the lightweight ULCNN. On the more challenging dataset, HisarMod2019.1, our model achieves an average accuracy of 43.97%, surpassing most models and closely approaching the performance of ULCNN (43.85%), while remaining competitive with PET-CGDNN. The performance of all models on HisarMod2019.1 is generally lower compared to that in the literature [2]. This is because the size of the training set used in this study is only 12.5% of the training set size reported in the literature [2]. In the field of deep learning, the amount of training data significantly impacts model performance. Typically, more training data enables the model to better learn the patterns and features within the data, thus improving its generalization ability on unseen data. Therefore, when the size of the training set is substantially reduced, the model may not be able to sufficiently learn the necessary features, resulting in decreased performance. Additionally, HisarMod2019.1 is designed to include multiple channel environments and a larger number of modulation categories, further increasing the complexity of the task.

When compared to other CVNNs, such as CDSN and CSDNN, our model exhibits significant advantages in parameter compression, computational efficiency, and generalization. In terms of model parameters, the proposed model achieves a two-order reduction compared to these CVNNs. For example, CDSN requires 1336.1 K/1331.6 K parameters on RML2016.10a and RML2016.10b, while CSDNN requires 327.1 K/315.4 K parameters. In contrast, our model requires only 9.0 K parameters, achieving reductions of 99.33% compared to CDSN and 97.25% compared to CSDNN. Furthermore, our model achieves FLOPs of 0.0006–0.00062 G/Sample on RML2016.10a and RML2016.10b, representing an 82.2% reduction compared to CDSN (0.00337 G/Sample) and a 93.5% reduction compared to CSDNN (0.00923 G/Sample). As shown in Figure 7 (g2–l2), compared to CDSN and CSDNN, our model achieves better data separation for different modulation categories at SNR = 2 dB. Notably, the data for QAM16 and QAM64 are relatively more compact and clustered, which facilitates subsequent classification tasks.

In addition, our model demonstrates superior generalization capabilities on RML2016.10b and HisarMod2019.1. It achieves an average accuracy of 63.97% on RML2016.10b, comparable to CDSN (64.04%) and CSDNN (65.82%), but with only 1.4% (vs. CDSN) and 2.8% (vs. CSDNN) of their parameter counts. And our model achieves an accuracy of 43.97% on HisarMod2019.1, improving by 37.3% over CDSN (32.04%) and by 1.47% over CSDNN (43.33%). The result highlights the effectiveness of the lightweight design of the proposed model in achieving high performance with minimal computational resources.

## 5. Conclusions

A lightweight dual-branch complex-valued neural network (LDCVNN) is proposed in this paper, which is capable of extracting features enriched with phase information as well as features possessing complex-scaling equivariance. The proposed model, with its compact design of only 9.0 K parameters, achieves the highest average accuracy on RML2016.10a without the need for data augmentation. It also achieves reductions of 99.33% compared to CDSN and 97.25% compared to CSDNN. Extensive evaluations conducted on other datasets, including RML2016.01b, RML2018.01a, and HisarMod2019, demonstrate that the proposed method successfully strikes a favorable balance between computational efficiency and classification performance. Compared to contemporary CVNNs employed for AMC, our approach exhibits superior lightweight properties and strong generalization capabilities. This research offers an effective solution for AMC tasks in resource-constrained scenarios, holding significant theoretical and practical implications.

For future work, we suggest exploring strategies to further enhance model performance under more challenging channel conditions. Additionally, investigating methods to enable continuous learning and real-time adaptation in dynamically evolving complex electromagnetic environments would allow the full potential of the model’s lightweight and efficient design to be realized.

## Figures and Tables

**Table 1:** AMR Open-source Datasets.

Dataset	RML2016.10a [36]	RML2016.10b [37]	RML2018.01a [38]	HisarMod2019.1 [39]
Details	classes *: 11 SNR: −20:2:18 dim: 2 × 128 size: 220 K	classes *: 10 SNR: −20:2:18 dim: 2 × 128 size: 1.2 M	classes *: 24 SNR: −20:2:30 dim: 2 × 1024 size: 2.56 M	classes *: 26 SNR: −20:2:18 dim: 2 × 1024 size: 780 K

* classes is the number of modulation types.

**Table 2:** LDCVNN Layer Details with Varying Input Lengths. (L1 = 128, L2 = 1024).

Layer	Input Shape		Kernel		Stride	Output Shape	
	L1	L2	L1	L2	-	L1	L2
wFM	[2,1,1,128]	[2,1,1,1024]	7	9	2	[2,16,1,61]	[2,16,1,508]
tReLU	[2,16,1,61]	[2,16,1,508]	-	-	-	[2,16,1,61]	[2,16,1,508]
wFM	[2,16,1,61]	[2,16,1,508]	7	9	1	[2,16,1,55]	[2,16,1,500]
tReLU	[2,16,1,55]	[2,16,1,500]	-	-	-	[2,16,1,55]	[2,16,1,500]
CDSC1d	[2,128]	[2,1024]	7	9	2	[32,61]	[32,508]
CBN + CReLU	[32,61]	[32,508]	-	-	-	[32,61]	[32,500]
CDSC1d	[32,61]	[32,500]	7	9	2	[32,61]	[32,500]
CBN + CReLU	[32,61]	[32,500]	-	-	-	[32,55]	[32,500]
weighted feature fusion	[2,16,1,55], [32,55]	[2,16,1,500], [32,500]	-	-	-	[32,55]	[32,500]
CBlock(Rep = 5)	[32,55]	[32,500]	3	3	2	[48,28]	[48,250]
CBlock(Rep = 1)	[48,28]	[48,250]	1	1	1	[48,28]	[48,250]
CAP	[48,28]	[48,250]	-	-	-	[48,1]	[48,1]
CFC	[48]	[48]	-	-	-	[classes *]	[classes *]

* classes is the number of modulation types.

**Table 3:** Main architectures of the compared models.

Model	Main Structure	Network Type
CNN2 [39]	CNN + DNN	RVNN
CLDNN [12]	CNN + LSTM + DNN	RVNN
CGDNN [40]	CNN + GRU + DNN	RVNN
ResNet [12]	ResNet	RVNN
MCLDNN [11]	CNN + LSTM + Multi-channel	RVNN
ICAMC [41]	CNN + Gaussian noise	RVNN
SCNN [42]	CNN	RVNN
PET-CGDNN [25]	CNN + GRU + DNN	PET + RVNN
ULCNN [32]	CV-CNN + GRU +DNN	DA + RVNN (with CV-CNN)
CDSN [19]	CV-CNN + CV-DNN	CVNN
CSDNN [33]	CV-CNN + Residual Connection + CDSC + CV-DNN	CVNN

**Table 4:** Effect of placing CSCConv at different positions.

Position	Capacity *	Average Accuracy
①	9025	62.41%
②	9977	61.40%
③	9977	61.43%

* Capacity is the number of model parameters.

**Table 5:** The ablation experiments on RML2016.10a.

Model	Ablation Part	Average Accuracy (All SNRs)	Capacity *
LDCVNN	-	62.41%	9025
LDCVNN-A	w/o CSEFE	61.89% (↓0.52%)	8332
LDCVNN-B	w/o PIEF	56.23% (↓6.18%)	8051
LDCVNN-C	w/o CSCConv	60.80% (↓1.61%)	8225
LDCVNN-D	w/o CBlock	61.53% (↓0.88%)	7609
LDCVNN + DA	-	62.46% (↑0.05%)	-

* Capacity is the number of model parameters.

**Table 6:** Performance metrics on RML2016.10a.

Model	Average Accuracy (All SNRs)	Average Accuracy (≥0 dB)	Highest Accuracy	F1-Score (%)	Capacity * (K)	FLOPs (G/Sample)	Test Time (ms/Sample)
CNN2	51.46%	74.85%	76.45%	47.99	858.1	0.07878	0.6350
CLDNN	59.53%	88.23%	89.36%	56.88	76.7	0.01991	0.1340
CGDNN	59.93%	89.48%	90.77%	57.59	52.0	0.00170	0.0454
ResNet	46.32%	71.34%	74.64%	42.33	3098.3	0.12413	0.8657
MCLDNN	60.83%	89.82%	90.77%	58.73	405.8	0.04869	0.1028
ICAMC	51.47%	75.34%	76.91%	53.09	1263.6	0.01466	0.0624
SCNN	55.45%	82.81%	84.36%	47.92	104.1	0.00189	0.0635
PET-CGDNN	60.85%	90.31%	91.55%	58.68	71.8	0.00831	0.0515
ULCNN	60.58%	90.11%	91.19%	58.65	9.4	0.00082	0.0605
CDSN	50.59%	74.82%	76.55%	49.29	1336.1	0.00923	0.0570
CSDNN	58.66%	86.91%	88.09%	56.90	327.1	1.1811	0.0787
Our Model	62.41%	91.63%	92.36%	59.58	9.0	0.00060	0.4210

* Capacity is the number of model parameters.

**Table 7:** Performance metrics on RML2016.10b.

Model	Average Accuracy (All SNRs)	Average Accuracy (≥0 dB)	Highest Accuracy	F1-Score (%)	Capacity * (K)	FLOPs (G/Sample)	Test Time (ms/Sample)
CNN2	48.74%	70.88%	71.98%	45.22	858.0	0.07878	0.6980
CLDNN	61.38%	89.41%	90.25%	59.70	76.4	0.01991	0.1403
CGDNN	63.71%	92.31%	92.90%	62.47	49.7	0.00170	0.0448
ResNet	47.65%	72.64%	75.59%	45.61	3098.2	0.12413	0.8758
MCLDNN	65.49%	93.50%	94.09%	64.31	405.7	0.04869	0.1103
ICAMC	59.85%	87.56%	88.67%	58.51	1263.5	0.01466	0.0590
SCNN	53.48%	76.76%	77.67%	50.62	95.9	0.00188	0.0582
PET-CGDNN	65.00%	93.30%	93.86%	64.30	71.6	0.00831	0.0508
ULCNN	63.09%	91.72%	92.46%	62.18	9.3	0.00082	0.0653
CDSN	64.04%	86.19%	87.08%	63.24	1331.6	0.00337	0.0606
CSDNN	65.82%	91.34%	91.73%	65.13	315.4	0.00923	0.0900
Our Model	63.97%	92.29%	93.18%	63.00	9.0	0.00062	0.4209

* Capacity is the number of model parameters.

**Table 8:** Performance metrics on RML2018.01a.

Model	Average Accuracy (all SNRs)	Average Accuracy (≥0 dB)	Highest Accuracy	F1-Score (%)	Capacity * (K)	FLOPs (G/Sample)	Test Time (ms/Sample)
CNN2	52.81%	76.15%	81.73%	50.78	1777.3	0.6302	2.5694
CLDNN	47.42%	69.03%	73.93%	44.90	80.0	0.1669	2.0549
CGDNN	39.29%	56.38%	58.61%	33.44	512.3	0.0151	1.8561
ResNet	41.95%	63.65%	70.65%	41.30	21450.0	0.9930	2.7234
MCLDNN	61.56%	90.04%	96.76%	60.11	407.5	0.4019	2.0184
ICAMC	47.21%	68.31%	72.39%	44.64	8605.3	0.1183	2.4862
SCNN	30.09%	41.22%	43.03%	24.81	1586.9	0.0160	2.4636
PET-CGDNN	61.01%	89.16%	96.21%	59.85	75.2	0.0719	1.8814
ULCNN	58.55%	85.40%	92.57%	57.17	9.9	0.0067	2.3571
CDSN	40.20%	56.75%	58.58%	37.06	1362.7	0.0120	3.4023
CSDNN	57.91%	84.19%	90.82%	56.27	333.8	0.0741	3.5972
Our Model	60.17%	88.91%	96.12%	59.18	11.2	0.0066	3.6759

* Capacity is the number of model parameters.

**Table 9:** Performance metrics on HisarMod2019.1.

Model	Average Accuracy (All SNRs)	Average Accuracy (≥0 dB)	Highest Accuracy	F1-Score (%)	Capacity * (K)	FLOPs (G/Sample)	Test Time (ms/Sample)
CNN2	36.31%	39.84%	40.54%	35.39	1777.6	0.6302	2.5212
CLDNN	39.82%	49.74%	51.50%	39.16	80.5	0.1669	1.9662
CGDNN	33.45%	37.45%	38.31%	32.67	552.7	0.0151	1.8690
ResNet	32.47%	35.82%	37.04%	31.91	21450.3	0.0210	2.7768
MCLDNN	43.09%	49.60%	51.65%	42.93	407.7	0.1452	2.0062
ICAMC	26.49%	29.02%	29.69%	24.26	8605.6	0.1183	2.3548
SCNN	30.89%	34.01%	34.54%	30.23	1718.0	0.0161	2.3327
PET-CGDNN	47.05%	52.13%	53.35%	46.77	75.5	0.0719	1.8153
ULCNN	43.85%	48.10%	49.77%	42.32	9.9	0.0054	2.3179
CDSN	32.04%	36.13%	37.23%	29.24	1360.3	0.0120	2.3788
CSDNN	43.33%	48.95%	50.35%	41.76	334.8	0.0741	2.4996
Our Model	43.97%	50.47%	53.08%	43.02	11.3	0.0067	2.5524

* Capacity is the number of model parameters.

## Data Availability

The original contributions presented in this study are included in the article. Further inquiries can be directed to the corresponding author.

## Abbreviations

The following abbreviations are used in this manuscript:

AMC	automatic modulation classification
LDCVNN	lightweight dual-branch complex-valued neural network
CVNN	complex-valued neural network
RVNN	real-valued neural network
IQ	in-phase and quadrature
SCConv	spatial and channel reconstruction convolution
CBlock	complex-valued depthwise separable convolution block
CDSC	complex-valued depthwise separable convolution
CR	cognitive radio
CNN	convolutional neural network
GRU	gated recurrent unit
DA	data augmentation
DSC	depthwise separable convolution
ReLU	rectified linear unit
DWC	depthwise convolution
SRU	spatial reconstruction unit
CRU	channel reconstruction unit
GN	group normalization
PIFE	phase information feature extraction
CSEFE	complex-valued scaling equivariant feature extraction
FEDR	feature extraction and dimension reduction
TP	true positive
FP	false positive
TN	true negative
FN	false negative
FLOPs	floating-point operations per second
DNN	dense neural network
LSTM	long short-term memory network
CConv	complex-valued convolution
wFM	weighted Fréchet mean filtering
tReLU	tangent rectified linear unit
CMP	complex-valued max pooling
CAP	complex-valued average pooling
CBN	complex-valued batch normalization
CFC	complex-valued full connection
CBlock	complex-valued depthwise separable convolution block
CDSC	complex-valued depthwise separable convolutions
CPWC	complex-valued pointwise convolution
CSCConv	complex-valued spatial and channel reconstruction convolution

## References

[1] Huynh-The T., Pham Q.V., Nguyen T.V., Nguyen T.T., Ruby R., Zeng M., Kim D.S. (2021). Automatic Modulation Classification: A Deep Architecture Survey. IEEE Access.

[2] Zhang F., Luo C., Xu J., Luo Y., Zheng F.-C. (2022). Deep learning based automatic modulation recognition: Models, datasets, and challenges. Digit. Signal Process..

[3] Dulek B. (2017). Online Hybrid Likelihood Based Modulation Classification Using Multiple Sensors. IEEE Trans. Wirel. Commun..

[4] Wen W., Mendel J.M. (2000). Maximum-likelihood classification for digital amplitude-phase modulations. IEEE Trans. Commun..

[5] Xu J.L., Su W., Zhou M. (2011). Likelihood-Ratio Approaches to Automatic Modulation Classification. IEEE Trans. Syst. Man Cybern. Part C (Appl. Rev.).

[6] Hazza A., Shoaib M., Alshebeili S.A., Fahad A. An overview of feature-based methods for digital modulation classification. Proceedings of the 2013 1st International Conference on Communications Signal Processing, and their Applications (ICCSPA).

[7] Meng F., Chen P., Wu L., Wang X. (2018). Automatic modulation classification: A deep learning enabled approach. IEEE Trans. Veh. Technol..

[8] Snoap J.A., Popescu D.C., Latshaw J.A., Spooner C.M. (2023). Deep-Learning-Based Classification of Digitally Modulated Signals Using Capsule Networks and Cyclic Cumulants. Sensors.

[9] Snoap J.A., Popescu D.C., Spooner C.M. (2024). Deep-Learning-Based Classifier With Custom Feature-Extraction Layers for Digitally Modulated Signals. IEEE Trans. Broadcast..

[10] Rajendran S., Meert W., Giustiniano D., Lenders V., Pollin S. (2018). Deep Learning Models for Wireless Signal Classification with Distributed Low-Cost Spectrum Sensors. IEEE Trans. Cogn. Commun. Netw..

[11] Xu J., Luo C., Parr G., Luo Y. (2020). A Spatiotemporal Multi-Channel Learning Framework for Automatic Modulation Recognition. IEEE Wirel. Commun. Lett..

[12] Liu X., Yang D., El Gamal A. Deep Neural Network Architectures for Modulation Classification. Proceedings of the 51st IEEE Asilomar Conference on Signals Systems, and Computers.

[13] Clarke T.L. Generalization of neural networks to the complex plane. Proceedings of the 1990 IJCNN International Joint Conference on Neural Networks.

[14] Trabelsi C., Bilaniuk O., Zhang Y., Serdyuk D., Subramanian S., Santos J.F., Mehri S., Rostamzadeh N., Bengio Y., Pal C.J. (2017). Deep Complex Networks. arXiv.

[15] Hirose A., Yoshida S., Lu B.-L., Zhang L., Kwok J. (2011). Comparison of Complex- and Real-Valued Feedforward Neural Networks in Their Generalization Ability. Neural Information Processing.

[16] Lee C., Hasegawa H., Gao S. (2022). Complex-Valued Neural Networks: A Comprehensive Survey. IEEE-CAA J. Autom. Sin..

[17] Xu J., Wu C., Ying S., Li H. (2022). The Performance Analysis of Complex-Valued Neural Network in Radio Signal Recognition. IEEE Access.

[18] Li W., Xie W., Wang Z., Zeng J., Jing W., Song X., Lu Z. (2020). Complex-Valued Densely Connected Convolutional Networks. Data Science.

[19] Tu Y., Lin Y., Hou C., Mao S. (2020). Complex-Valued Networks for Automatic Modulation Classification. IEEE Trans. Veh. Technol..

[20] Kim S., Yang H.-Y., Kim D. (2022). Fully Complex Deep Learning Classifiers for Signal Modulation Recognition in Non-Cooperative Environment. IEEE Access.

[21] Cheng R., Chen Q., Huang M. (2024). Automatic modulation recognition using deep CVCNN-LSTM architecture. Alex. Eng. J..

[22] Chakraborty R., Wang J., Yu S.X. (2019). SurReal: Fréchet Mean and Distance Transform for Complex-Valued Deep Learning. arXiv.

[23] Chakraborty R., Xing Y., Yu S.X. (2020). SurReal: Complex-Valued Learning as Principled Transformations on a Scaling and Rotation Manifold. IEEE Trans. Neural Netw. Learn. Syst..

[24] Singhal U., Xing Y., Yu S.X. (2021). Co-domain Symmetry for Complex-Valued Deep Learning. arXiv.

[25] Zhang F., Luo C., Xu J., Luo Y. (2021). An Efficient Deep Learning Model for Automatic Modulation Recognition Based on Parameter Estimation and Transformation. IEEE Commun. Lett..

[26] Lin Y., Tu Y., Dou Z. (2020). An Improved Neural Network Pruning Technology for Automatic Modulation Classification in Edge Devices. IEEE Trans. Veh. Technol..

[27] Tu Y., Lin Y. (2019). Deep Neural Network Compression Technique Towards Efficient Digital Signal Modulation Recognition in Edge Device. IEEE Access.

[28] Zhang X., Zhao H., Zhu H., Adebisi B., Gui G., Gacanin H., Adachi F. (2022). NAS-AMR: Neural Architecture Search-Based Automatic Modulation Recognition for Integrated Sensing and Communication Systems. IEEE Trans. Cogn. Commun. Netw..

[29] Fu X., Gui G., Wang Y., Ohtsuki T., Adachi F. (2021). Lightweight Automatic Modulation Classification Based on Decentralized Learning. IEEE Trans. Cogn. Commun. Netw..

[30] Lin Y., Zha H., Tu Y., Zhang S., Yan W., Xu C. (2023). GLR-SEI: Green and Low Resource Specific Emitter Identification Based on Complex Networks and Fisher Pruning. IEEE Trans. Emerg. Top. Comput. Intell..

[31] Wang F., Shang T., Hu C., Liu Q. (2023). Automatic Modulation Classification Using Hybrid Data Augmentation and Lightweight Neural Network. Sensors.

[32] Guo L., Wang Y., Liu Y., Lin Y., Zhao H., Gui G. (2024). Ultralight Convolutional Neural Network for Automatic Modulation Classification in Internet of Unmanned Aerial Vehicles. IEEE Internet Things J..

[33] Xiao C., Yang S., Feng Z. (2023). Complex-Valued Depthwise Separable Convolutional Neural Network for Automatic Modulation Classification. IEEE Trans. Instrum. Meas..

[34] Fréchet M.R. (1948). Les éléments aléatoires de nature quelconque dans un espace distancié. Ann. L’institut Henri Poincaré.

[35] Li J., Wen Y., He L. SCConv: Spatial and Channel Reconstruction Convolution for Feature Redundancy. Proceedings of the 2023 IEEE/CVF Conference on Computer Vision and Pattern Recognition (CVPR).

[36] O’Shea T.J., West N. Radio Machine Learning Dataset Generation with GNU Radio. Proceedings of the GNU Radio Conference.

[37] O’Shea T.J., Corgan J., Clancy T.C. Convolutional radio modulation recognition networks. Proceedings of the Engineering Applications of Neural Networks: 17th International Conference, EANN 2016.

[38] O’Shea T.J., Roy T., Clancy T.C. (2018). Over-the-Air Deep Learning Based Radio Signal Classification. IEEE J. Sel. Top. Signal Process..

[39] Tekbıyık K., Ekti A.R., Görçin A., Kurt G.K., Keçeci C. Robust and Fast Automatic Modulation Classification with CNN under Multipath Fading Channels. Proceedings of the 2020 IEEE 91st Vehicular Technology Conference (VTC2020-Spring).

[40] Njoku J.N., Morocho-Cayamcela M.E., Lim W. (2021). CGDNet: Efficient Hybrid Deep Learning Model for Robust Automatic Modulation Recognition. IEEE Netw. Lett..

[41] Hermawan A.P., Ginanjar R.R., Kim D.S., Lee J.M. (2020). CNN-Based Automatic Modulation Classification for Beyond 5G Communications. IEEE Commun. Lett..

[42] Zeng Y., Zhang M., Han F., Gong Y., Zhang J. (2019). Spectrum Analysis and Convolutional Neural Network for Automatic Modulation Recognition. IEEE Wirel. Commun. Lett..

